# Expression of a Cystatin Transgene in Eggplant Provides Resistance to Root-knot Nematode, *Meloidogyne incognita*

**DOI:** 10.3389/fpls.2016.01122

**Published:** 2016-07-28

**Authors:** Pradeep K. Papolu, Tushar K. Dutta, Nidhi Tyagi, Peter E. Urwin, Catherine J. Lilley, Uma Rao

**Affiliations:** ^1^Division of Nematology, ICAR-Indian Agricultural Research InstituteNew Delhi, India; ^2^SRM UniversityChennai, India; ^3^Centre for Plant Sciences, University of LeedsLeeds, UK

**Keywords:** OC-IΔD86, TUB-1, eggplant, multiplication factor, proteinase inhibitor

## Abstract

Root-knot nematodes (RKN) cause substantial yield decline in eggplant and sustainable management options to minimize crop damage due to nematodes are still limited. A number of genetic engineering strategies have been developed to disrupt the successful plant–nematode interactions. Among them, delivery of proteinase inhibitors from the plant to perturb nematode development and reproduction is arguably the most effective strategy. In the present study, transgenic eggplant expressing a modified rice cystatin (OC-IΔD86) gene under the control of the root-specific promoter, TUB-1, was generated to evaluate the genetically modified nematode resistance. Five putative transformants were selected through PCR and genomic Southern blot analysis. Expression of the cystatin transgene was confirmed in all the events using western blotting, ELISA and qPCR assay. Upon challenge inoculation, all the transgenic events exhibited a detrimental effect on RKN development and reproduction. The best transgenic line (a single copy event) showed 78.3% inhibition in reproductive success of RKN. Our results suggest that cystatins can play an important role for improving nematode resistance in eggplant and their deployment in gene pyramiding strategies with other proteinase inhibitors could ultimately enhance crop yield.

## Introduction

Eggplant (*Solanum melongena* L.), popularly known as brinjal or aubergine, is consumed as a staple food in Asia and the Mediterranean region with China and India contributing the maximum share of global production (source^1^). Due to its high nutritive value this solanaceous vegetable is often recommended to tackle malnutrition problems^2^. Eggplant farmers suffer substantial yield losses due to the attack of various pests and diseases including nematodes. Among nematodes, root-knot nematode (RKN: *Meloidogyne incognita*) is considered the major yield constraint for eggplant (Di Vito et al., 1986; Koenning et al., 1999; Goggin et al., 2006; Bagnaresi et al., 2013). In India, according to a conservative estimate, RKN causes 16.67% yield decline in brinjal which translates into almost 23$ million annual monetary loss (Jain et al., 2007). RKNs are soil borne endoparasites that cause significant damage in crop plants in terms of severe galling of roots, stunted growth of plants due to reduced nutrient uptake from soil and predisposition of roots to secondary pathogen invasion (Jones et al., 2013). Infective second stage juveniles (J2s) penetrate the root intercellularly and induce the formation of specialized feeding cells (giant cells) in the developing vascular region of the root apex. The J2 matures into the sedentary adult and reproduces parthenogenetically to complete its life cycle, sustained by the nutrients provided by the giant cells. Root tissues surrounding the giant cells are hypertrophied to form the root galls (Williamson and Gleason, 2003).

To date, very few management options are available to growers to circumvent the RKN problem in eggplant. Cultural methods such as soil solarization, crop rotation with non-host crops, summer plowing, etc., warrant a lengthy fallow period which is not practically feasible for the farming community who may lose cropping opportunities during that period. No potent biological control method has been standardized yet to reduce RKN populations below the threshold level in eggplant. Chemical nematicides are continually being withdrawn from the market owing to the concerns of groundwater contamination and residue toxicity to humans. As the resistance breeding approach requires longer time (feasibility of plant grafting on resistant rootstock for RKN resistance is yet to be explored) and R-gene sources are still not known in eggplant, transgenic resistance to *M. incognita* offers an attractive alternative.

One approach to increase nematode resistance in crop plants is the transgenic expression of anti-nematode proteins or proteinase inhibitors that disrupt protein digestion in the nematodes leading to their arrested development in host plants (Fuller et al., 2008). Cystatins (cysteine proteinase inhibitors) are low molecular weight proteins which are found in a number of plant species where they can ostensibly act as potent inhibitors of exogenous proteases, such as digestive enzymes of invasive pests and pathogens (Samac and Smigocki, 2003; Benchabane et al., 2010; Martinez et al., 2012) in addition to roles as regulators of endogenous enzymes (Benchabane et al., 2010). Oryzacystatins (OC-I and OC-II) and a genetically altered cystatin (OC-IΔD86, a variant of wild-type (WT) OC-I with a deletion of an aspartic acid residue at position 86) have been used to engineer a range of plant species (*Arabidopsis*, rice, potato, tomato, alfalfa, banana, sweet potato, and lily) that displayed resistance against nematodes with diverse feeding habits, such as root-knot (*M. incognita, M. hapla*) and cyst nematodes (*Heterodera schachtii, Globodera pallida*), reniform nematode (*Rotylenchulus reniformis*), stem nematode (*Ditylenchus destructor*), root lesion nematode (*Pratylenchus penetrans*) and burrowing nematode (*Radopholus similis*; Urwin et al., 1995, 1997, 1998, 2000; Vain et al., 1998; Samac and Smigocki, 2003; Atkinson et al., 2004a; Lilley et al., 2004; Chan et al., 2010; Gao et al., 2011; Vieira et al., 2015). Overexpression of a maize cystatin (CC-II) in banana substantially reduced infection by *R. similis* and *Helicotylenchus multicinctus* (Roderick et al., 2012). In addition, successful field trials of transgenic potato (Urwin et al., 2001, 2003) and banana (Tripathi et al., 2015) expressing cystatin reinforced the efficacy of proteinase inhibitors for engineering nematode resistance in crop plants.

The majority of the studies cited above have used constructs providing constitutive expression under the control of the promoter CaMV35S (cauliflower mosaic virus 35S). However, to minimize the exposure of non-target organisms to proteinase inhibitors, using a promoter that predominantly expresses in the root system is always advantageous. Green et al. (2002) demonstrated that the TUB-1 promoter derived from the β-tubulin gene of *Arabidopsis thaliana* effectively delivered the designed construct to *M. incognita* from rice. Similarly, potato plants expressing a modified cystatin (OC-IΔD86) transgene under the control of the TUB-1 promoter displayed the highest level of resistance to *M. incognita* compared to other root-specific promoters tested. Along with that, the level of resistance obtained with the TUB-1 promoter was comparable with that when using the CaMV35S promoter (Lilley et al., 2004).

Earlier, transgenic wheat expressing a potato serine protease inhibitor exhibited resistance to cereal cyst nematode, *H. avenae* in correlation with increased plant yield (Vishnudasan et al., 2005). However, in *M. incognita* metallo and cysteine proteases are the most abundant proteolytic enzymes followed by serine, aspartic and threonine proteases (Castagnone-Sereno et al., 2011). Cathepsin L-like cysteine proteinases play a profound role in the digestion process of *M. incognita* (Neveu et al., 2003; Shingles et al., 2007).

Genetic engineering for resistance to *M. incognita* in eggplant may have important economic and social impacts in India, Asia, the Mediterranean region and other eggplant producing countries worldwide, such as Brazil (Pinheiro et al., 2013). Therefore, in the present study, a cystatin (OC-IΔD86) transgene under the control of the TUB-1 promoter was expressed in eggplant to evaluate the effect of the transgenic plants on the development and reproduction of *M. incognita* in contained growth conditions. Significant findings from this study demonstrate the potential of proteinase inhibitors to manage RKN problems in eggplant at the field level, thereby increasing the yield of this important vegetable crop.

## Materials and Methods

### Plant Transformation

**FIGURE 1:**
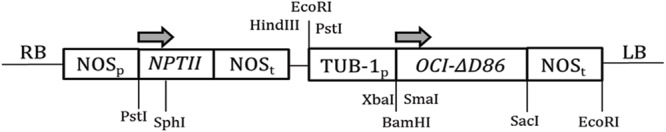
**Schematic representation of the T-DNA region of the transformation construct.** The tubulin promoter (TUB-1_p_) and nopaline synthase terminator (NOS_t_) drives the expression of OCI-ΔD86. The kanamycin resistance gene neomycin phosphotransferase (*nptII*) was used as the selectable marker located within left (LB) and right (RB) borders of T-DNA. Arrows indicate direction of transcription.

A derivative of the binary vector pBI121 containing the expression cassette of OC-IΔD86 modified to potato codon usage under the control of the *Arabidopsis* TUB-1 promoter (**Figure 1**), (Lilley et al., 2004) was introduced into competent *Agrobacterium tumefaciens* strain LBA4404 by electroporation as described by Shen and Forde (1989). Subsequently, leaf disks of *S. melongena* cv. Pusa Purple Long were transformed as described by Papolu et al. (2013) and Dutta et al. (2015). Transgenic eggplants were rooted in Murashige and Skoog (MS) medium supplemented with 0.1 mg/L NAA (naphthalene acetic acid) before transfer to soil. Plants transformed with *Agrobacterium* that did not harbor the pBI construct, and hence underwent no kanamycin selection, served as the control.

### Detection of Transgene Using PCR, qPCR, and Southern Hybridization

Genomic DNA was extracted from fresh *in vitro* plants using a Nucleospin Plant II DNA extraction kit (Macherey-Nagel, Germany) following the manufacturer’s instructions. For PCR-based detection, gene-specific primers (OC-IΔD86 forward 5′-TCAGACGGAGGACCAGTTTT-3′ and OC-IΔD86 reverse 5′-CATCCATGGTTTTTCCCAAA-3′) and primers specific for the kanamycin resistance gene (nptII forward 5′-CAATCGGCTGCTCTCATGCCG-3′ and nptII reverse 5′-AGGCGATAGAAGGCGATGCGC-3′) were used.

Total RNA was isolated from roots of fresh *in vitro* plants using a Nucleospin Plant II RNA extraction kit (Macherey-Nagel, Germany) following the manufacturer’s instructions. Approximately 400 ng of purified RNA was reverse transcribed to cDNA using a cDNA synthesis kit (Superscript VILO, Invitrogen). Quantitative RT-PCR (qPCR) amplification to assess the level of OC-IΔD86 expression in eggplant root was performed using specific primers (OC-IΔD86 RT-forward 5′-CGAACCAGTTGGAAATGAAA-3′ and OC-IΔD86 RT-reverse 5′-AAGTAGTACAAAGTTCCAGCAACAA-3′) with the following PCR conditions: a hot start of 95°C for 5 min; 40 cycles of 95°C for 15 s and 60°C for 1 min. In order to ascertain the specificity of amplification, a melt curve analysis or dissociation program was run as 95°C for 15 s; 60°C for 15 s followed by a slow ramp from 60 to 95°C. qPCR was performed in a realplex^2^ thermal cycler (Eppendorf, Germany) using SYBR Green PCR master-mix kit (Eurogentec). Gene expression was normalized using 18S rRNA (Gantasala et al., 2013) as reference. Relative differences in expression were analyzed through 2^-ΔΔ*C*_T_^ method (Livak and Schmittgen, 2001) after incorporating the data from three independent experiments, i.e., three separate plants from the same transgenic line.

In order to determine the integration pattern of *OC-IΔD86* and *nptII* transgenes in transgenic plants, genomic Southern hybridization was performed with PCR-confirmed events. Ten micrograms of isolated genomic DNA of each event was digested with BamHI enzyme (New England Biolabs), electrophoresed in a 0.8% agarose gel and transblotted to nitrocellulose membrane (BioRad Zeta Probe). For probing, a 250 bp fragment of the *Oc-IΔD86* gene and a 750 bp fragment of the *nptII* gene were used independently. Probe labeling, hybridization and blot development were carried out as described in Papolu et al. (2013) and Dutta et al. (2015).

### Detection of Cystatin Expression in Eggplant Using Western Blot and ELISA

Whole-protein extract from roots (100 mg) of fresh *in vitro* plants was collected in 1 ml of lysis buffer (Sigma, Germany) containing a protease inhibitor cocktail (Roche, Switzerland) according to the manufacturer’s instructions. The concentration of total soluble protein (TSP) was measured using Bio-Rad DC Protein Assay (Bio-Rad, USA).

Production of OC-IΔD86 protein in the transgenic eggplant lines was confirmed by western blotting. Each sample with total volume of 20 μl (containing 5 μg total protein extract) were separated on a 12% SDS-PAGE gel followed by transfer to a polyvinylidene fluoride (PVDF) membrane using standard equipment and protocols. The membrane blot was probed with primary rabbit polyclonal antibody raised against OC-IΔD86 protein at 1:5000 dilution followed by probing with anti-rabbit IgG/alkaline phosphatase conjugate (Sigma) at 1:2000 dilution. The signal was detected by colorimetric reaction with NBT/BCIP as substrates.

ELISA was conducted to determine the relative levels of OC-IΔD86 protein in roots of transgenic plants. The wells of a microtitre plate were coated with three technical replicates of extracted proteins (5 μg each diluted in 20 μl blocking solution) of WT and transformed plants, and a standard protein (OC-IΔD86 expressed in *E. coli*) at 4°C overnight. Post three washes with PBST (PBS containing 0.05% Tween 20) the plate was incubated with primary rabbit polyclonal antibody against OC-IΔD86 at 1:10,000 dilution followed by incubation with alkaline phosphatase conjugated with a goat anti-rabbit IgG (supplied by Genei, Bangalore) at 1:2000 dilution. Addition of *p*-nitrophenyl phosphate (pNPP) started the color reaction while addition of 2N H_2_SO_4_ terminated the reaction. The absorbance at each well was measured at 405 nm in a microplate reader (Biotek ELX800). Each line was measured twice in independent experiments. Quantitative analysis of TSP was performed according to Bradford (1976).

### Bioefficacy Analysis of Eggplants Expressing OC-IΔD86 against *M. incognita*

T_1_ seeds from the primary transgenic events (T_0_) were surface sterilized and germinated in MS medium containing kanamycin (100 mg L^-1^). Plants with well-developed root and shoot were then transferred to pots filled with autoclaved soil. Presence of *OC-IΔD86* and *nptII* genes were detected in the transformed plants using molecular analysis as described above. T_1_ plants were allowed to self-pollinate and T_2_ seeds were obtained. Homozygous T_2_ plants containing single and double copy insertions of *OC-IΔD86* were subjected to bioefficacy studies against *M. incognita*.

A pure culture of *M. incognita* was maintained on eggplant (cv. Pusa Purple Long) in the greenhouse. Egg masses were collected from the roots of 8-week-old plants using sterilized forceps and were kept for hatching in a double-layered tissue paper supported on a molded sieve of wire gauze in a Petri dish containing tap water (Southey, 1986).

The roots of 15 days old T_2_ plants [growing in 500 ml pots containing equal mixture of soil and soilrite (Keltech Energies Ltd., Bengaluru)] were inoculated with approximately 300 freshly hatched J2s in the vicinity of the root zone. RKN-infected T_2_ plants were grown at 28°C, 70% relative humidity and 16:8 h light:dark photoperiod in confinement. At 30 days post inoculation (DPI) plants were harvested, roots were washed free of soil and fresh root and shoot weight was recorded. Total number of galls, females, egg masses and eggs/egg mass for each plant were counted. In order to determine the effect of the proteinase inhibitor on nematode reproductive potential, the multiplication factor (MF) of *M. incognita* was calculated [(number of egg masses × number of eggs per egg mass) ÷ nematode inoculum level] for each transgenic event. Data were compared with those for WT plants challenged with a similar number of *M. incognita* J2s, grown under identical conditions. The infection bioassay was carried out as a randomized complete block design with six replicates. In addition, at 2 DPI, some of the plants from each treatment were harvested and roots were stained with acid fuchsin (Byrd et al., 1983) to record the number of J2s that had penetrated the root. Data were subjected to one-way ANOVA test followed by Tukey’s multiple-comparison test with significance level at *P* < 0.01 using SAS software (version 9.3).

## Results

### Molecular Characterization of Transgenic Eggplant Containing Cystatin Construct

**FIGURE 2:**
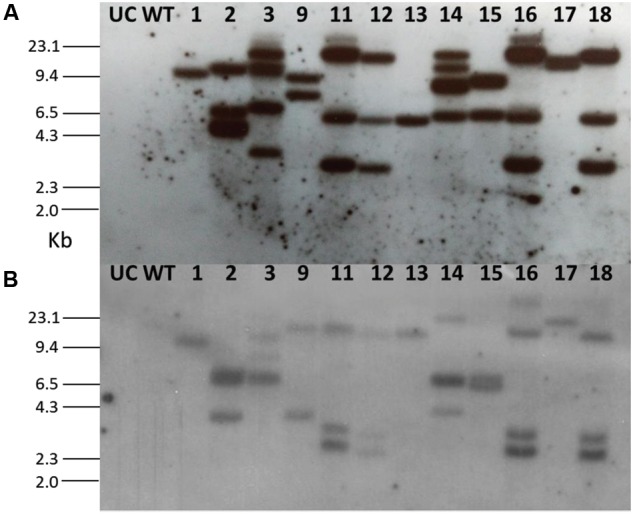
**Southern blot for T_0_ eggplant transformants containing the cystatin construct.** Probes **(A)** OC-IΔD86 and **(B)** nptII were used for hybridization. Untransformed control (UC) and wild-type (WT) plants did not show any hybridization signal.

Using the leaf disk*-Agrobacterium* co-cultivation method *A. tumefaciens* strain LBA4404 harboring the cystatin construct was used to transform eggplant (Supplementary Figure S1). After kanamycin selection and regeneration, 35 independent transgenic events (T_0_) were generated. Each event was genotyped through PCR and amplified fragments of both *OC-IΔD86* (256 bp) and *nptII* (750 bp) were detected in 24 out of 35 events (Supplementary Figure S2). Untransformed control plants and transgenic plants did not differ in morphological characteristics (data not shown). A total of 12 T_0_ lines (1, 2, 3, 9, 11, 12, 13, 14, 15, 16, 17, and 18) were subjected to Southern blot assay to analyze the integration patterns of both *OC-IΔD86* and *nptII* transgenes. Remarkably, an identical pattern of integration was observed for both *OC-IΔD86* and *nptII* transgenes in four of the selected events (11, 12, 16, and 18). Single copy insertion of T-DNA was observed in event numbers 1, 13, and 17, while double copy insertion was documented for event numbers 9 and 15, and the rest showed multiple copy insertions (**Figure 2**). Transgenic lines 1, 13, 17 (single copy events), 9, and 15 (double copy events) were used for further study.

After selecting the progeny plants through kanamycin, selected T_1_ and T_2_ events were genotyped through PCR (Supplementary Figure S2). Segregation frequencies for single copy events indicated that *OC-IΔD86* and *nptII* were integrated at one locus (3:1 after self-pollination) following a Mendelian pattern of inheritance (data not shown). Southern hybridization in T_2_ plants for *OC-IΔD86* transgene indicated that, all the progeny plants tested for events 13 and 17 and two progeny plants out of four in event 1 reaffirmed the single copy integration pattern (Supplementary Figure S3). However, in approximately 50% progeny plants of double copy events (9 and 15) the possibility of segregation distortion could not be ruled out (Supplementary Figure S3). To this end, five homozygous lines (1.1, 13.1, 17.1, 9.2, and 15.2) representing all the single and double copy events were used for subsequent studies.

### Expression of OC-IΔD86 in Transgenic Lines of Eggplant

**FIGURE 3:**
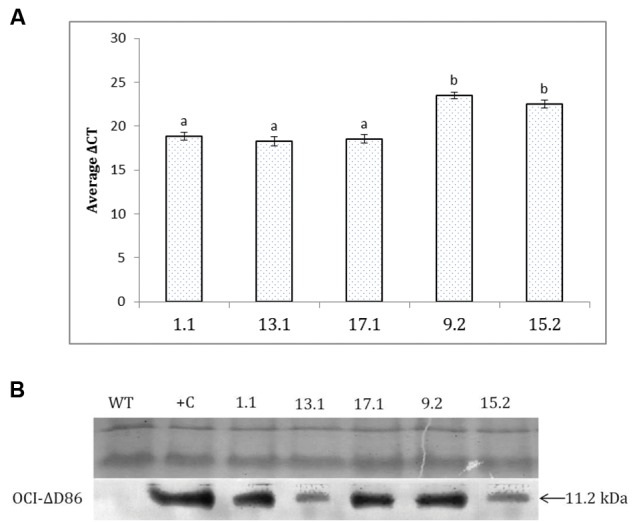
**Detection of cystatin expression in T_2_ events of eggplant by qPCR and western blot. (A)** Relative transcript levels of Oc-IΔD86 in different events are expressed as Δ*C*_t_ values which denotes the difference in Ct mean of Oc-IΔD86 and the reference gene (*18S rRNA* gene of eggplant). Higher values represent lower expression of Oc-IΔD86. Each bar represents mean ± SEM derived from three independent biological and three technical replicates. Bars with different letters are statistically different at *P* < 0.05. **(B)**. Total protein extracted from the roots of WT and transgenic lines (1.1, 13.1, 17.1, 9.2, and 15.2) were assayed with anti-OC-IΔD86 polyclonal antibody. Top panel demonstrates the equal loading of protein samples in SDS-PAGE. Positive control (+C) corresponds to WT extract spiked with 0.1% TSP of recombinant OC-IΔD86 (11.2 kDa). WT plants did not show any hybridization signal.

Using a qPCR assay, amplified transcripts of OC-IΔD86 were observed in all the T_2_ transgenic events. On the contrary, no transcripts corresponding to the cystatin transgene were detected in the RNA isolated from non-transgenic control plants. The data for the transgenic lines are therefore simply presented relative to the normaliser gene. Since the mean *C*_t_ of OC-IΔD86 was greater than the mean *C*_t_ of the normalizer gene, *18S rRNA*, for each transgenic line, significantly greater Δ*C*_T_ values (difference between mean *C*_t_ of OC-IΔD86 and 18S rRNA) in double copy events (9.2 and 15.2) indicated the lower quantitative expression of OC-IΔD86 in these events than the single copy ones (1.1, 13.1, and 17.1; **Figure 3A**). However, OC-IΔD86 expression was undetectable in any transgenic plant parts except root tissues. Western blotting assay revealed that a 11.2 kDa polypeptide, the expected molecular size of OC-IΔD86 protein, was specifically recognized by the anti-OC-IΔD86 antibody. This polypeptide only appeared in the transgenic lines 1.1, 13.1, 17.1, 9.2, and 15.2, but not in the WT plants (**Figure 3B**).

To quantitatively estimate the amount of OC-IΔD86 in transgenic T_2_ plants ELISA was conducted. Purified recombinant OC-IΔD86 protein produced in *E. coli* with known concentration was used as the standard. Results showed that the OC-IΔD86 expression level ranged between 0.09 to 0.15% TSP in different events. The expression level of OC-IΔD86 protein was highest (0.15 ± 0.04% TSP) in event 1.1. Expression of cystatin was not detected in WT plants (Supplementary Figure S4).

### Evaluation of T_2_ Events for Resistance to *M. incognita*

No significant difference was observed in the root mass between WT plants grown on non-selective medium and T_2_ plants grown on selection medium containing kanamycin (Supplementary Figure S5), indicating that neither the antibiotic marker nor the cystatin expression affected the root growth of transgenic plants. Additionally, there was no significant difference in the penetration ability of *M. incognita* J2 among WT and transgenic plants at 2 DPI (Supplementary Figure S6), suggesting that OC-IΔD86 did not inhibit RKN invasion.

**FIGURE 4:**
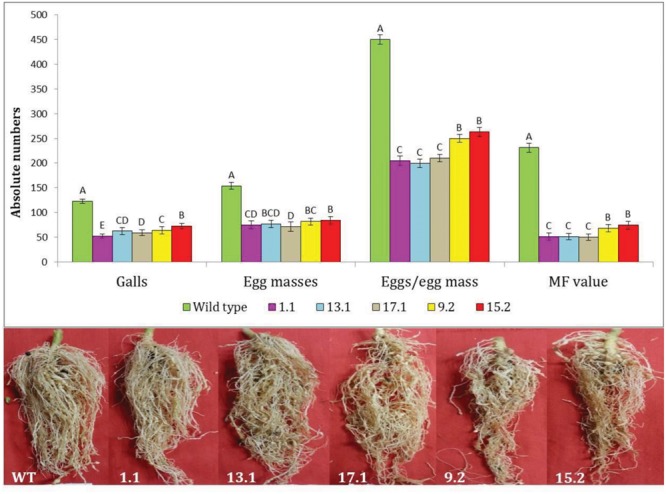
**Effect of cystatin overexpression in eggplant on development and reproduction of *M. incognita*.** Absolute numbers of galls, egg masses, eggs/egg mass, and the corresponding multiplication factor (MF) of *M. incognita* in different T2 events (1.1, 13.1, 17.1, 9.2, and 15.2) and WT plants at 30 DPI. Each bar represents the mean ± SE of *n* = 6, and bars with different letters (within each parameter) denote a significant difference at *P* < 0.01, Tukey’s test.

**FIGURE 5:**
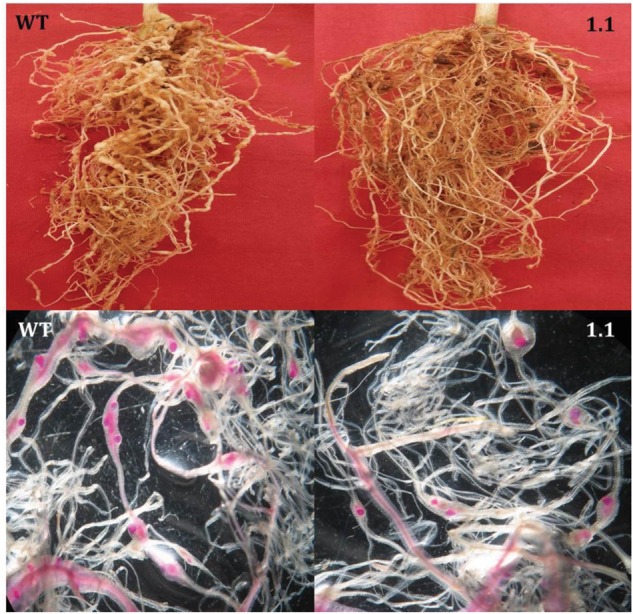
**Comparative infection of *M. incognita* in the roots of transgenic (a single copy T_2_ event, 1.1) and WT eggplant at 30 DPI. (Top)** Indicates that galling intensity was higher in WT roots than in the transgenic roots. **(Bottom)** Indicates that transgenic plants supported fewer female nematodes than the WT plants. Females inside the root were stained with acid fuchsin (Byrd et al., 1983).

Following *M. incognita* invasion, galls were induced in the roots of WT and transgenic plants (**Figure 4**). However, the number of root galls was considerably reduced in transgenic plants. At 30 DPI, the average number of galls per plant was significantly (*P* < 0.01) reduced by 40.81–57.22% in all cystatin overexpressing lines (1.1, 13.1, 17.1, 9.2, and 15.2) compared to the WT (**Figures 4** and **5**). Accordingly, a marked reduction (45.02–53.46%) in the number of egg masses was recorded in T_2_ plants compared to WT ones (**Figure 4**). Since each RKN female produces its progeny in a single egg mass, the number of egg masses also indicates the identical number of successfully reproducing females. Hence, plant-mediated delivery of OC-IΔD86 to post-parasitic juveniles of *M. incognita* resulted in perturbed development of juveniles to adult females and eventually, reduction in root galling was documented in transgenic plants (**Figure 5**). Nevertheless, mature females extracted from transgenic and non-transgenic plants were similar in shape and size (data not shown).

When the fecundity of *M. incognita* was measured in terms of eggs per egg mass in cystatin overexpressing lines, a significant (*P* < 0.01) reduction in the range of 41.48–55.70% was observed in those lines compared to WT plants (**Figure 4**). Given the ultimate importance of multiplication factor (MF) which is indicative of reproductive fitness and parasitic success of a nematode on host plants, our results demonstrated that MF was dramatically reduced by 67.81–78.30% in transgenic lines compared to WT plants. Interestingly, single copy transformants (1.1, 13.1, and 17.1) exhibited greater reduction in MF than double copy transformants (9.2 and 15.2). However, within the single or double copy transformants no significant (*P* < 0.01) difference was observed (**Figure 4**). The complete experiment was conducted twice and showed similar results on each occasion.

## Discussion

Cysteine proteinases play a major role in the digestion process of RKNs and binding of phytocystatins to the active sites of these proteinases can inhibit their activity, which in turn may affect nematode proteolytic digestion (Koritsas and Atkinson, 1994; Neveu et al., 2003; Shingles et al., 2007). Both engineered and native cystatins have provided resistance to a broad spectrum of phytonematodes in various host plants (Fuller et al., 2008; Chan et al., 2010; Gao et al., 2011; Vieira et al., 2015). Considering that cysteine proteinases are the only class of proteinases that are not expressed in the digestive system of mammals (Atkinson et al., 1995), cystatins offer an attractive option for formulating safer transgenic defense strategies against nematodes. Transgenic expression of OC-IΔD86 (a protein engineered variant of OC-I) seems to confer higher level of nematode resistance than the full length Oryzacystatin-I (OC-I) gene (Urwin et al., 1995; Vieira et al., 2015). Hence, in the present study, we have used the *OC-IΔD86* gene for generating transgenic eggplant and its inhibitory effect on RKN development and reproduction was evaluated. Tissue-specific promoters minimize the exposure of non-target organisms to cystatins and can efficiently deliver them into actively feeding nematodes (Samac and Smigocki, 2003). In accordance with this presumption, we have expressed the *OC-IΔD86* gene under the control of the TUB-1 promoter (Green et al., 2002; Lilley et al., 2004) to confine its expression to the RKN-inducible giant cells.

After successful transformation of eggplant with the OC-IΔD86 construct, five transgenic T_2_ events (1.1, 13.1, 17.1, 9.2, and 15.2) containing both single and double copy insertions of the *OC-IΔD86* transgene were selected for further studies. Subsequently, roots of plants from all the five events showed expression of OC-IΔD86 that varied among different T_2_ lines exemplifying that the transgenes were randomly integrated at various transcriptionally active sites in the eggplant genome. However, no correlation was observed between the level of OC-IΔD86 expression and the level of nematode resistance achieved.

Our data from independent nematode challenge assays indicate that although the level of resistance to RKN was greater in single copy T_2_ events than the double copy ones, a consistent level of resistance was achieved within the single or double copy events. The comparatively lower resistance of double copy events to *M. incognita* may be explained by the possibility that transgenes may co-suppress others in the vicinity in a homology-dependent manner. Similar silencing between transgenes and endogenous genes with a high level of homology has been reported in several plant species (Napoli et al., 1990). Our qPCR data indicated the relatively greater expression of OC-IΔD86 in single copy events than the double copy ones.

Overexpression of OC-IΔD86 caused a pronounced reduction in gall numbers in transgenic eggplant compared to WT plants. The possibility of reduced penetration leading to less gall formation in transgenic plants may be excluded since RKNs had invaded transgenic and non-transgenic plants alike in our experiments. Hence, OC-IΔD86 did not function as an anti-invasion agent; rather it had affected nematode development and reproduction in transgenic plants. In line with this assumption, fecundity of RKN in terms of numbers of egg masses (reflecting identical number of female nematodes) and eggs per egg mass was greatly reduced in transgenic plants compared to WT in our study. However, in contrast to studies of Atkinson et al. (1996) and Urwin et al. (1997), who reported the arrested growth of *M. incognita* in *OC-I*-expressing tomato hairy roots and *OC-IΔD86*-expressing *Arabidopsis*, respectively, no growth retardation of *M. incognita* females was documented in our study. Our revelation is in line with the findings of Chan et al. (2010) who expressed *Colocasia esculenta* cysteine proteinase inhibitor in tomato to impart resistance against *M. incognita*.

Nematode multiplication factor, which determines the parasitic success of a nematode in host plants, was reduced by 77.8–78.3% in single copy events and 67.8–70.5% in double copy events compared to WT plants. Our results are in agreement with the earlier studies of OC-IΔD86 cystatin for genetically modified nematode resistance. A reduction of 35% in female numbers and 69% in fecundity led to the corresponding 81% decrease in overall reproductive success of the sedentary endoparasite, *R. reniformis*, in *OC-IΔD86*-expressing *Arabidopsis* plants (Urwin et al., 2000). Low level expression of OC-IΔD86 in rice decreased the reproductive success of *M. incognita* by 55% (Vain et al., 1998). Banana plants expressing the same cystatin displayed 70% resistance to the migratory endoparasite, *R. similis* (Atkinson et al., 2004a). A partial resistance of 67% was achieved against *M. incognita* while expressing OC-IΔD86 in potato roots under the control of the TUB-1 promoter (Lilley et al., 2004).

Since it is practically improbable to achieve full control of nematode populations, partial resistance provided by a plant that limits nematode multiplication to a level at which damaging population densities do not build up is desirable. RKN species typically complete three generations throughout the growth of an annual host crop. Therefore, 60% control per generation is sufficient to bring down the RKN population below the economic threshold level. Accordingly, independently acting defenses over two generations, each providing 80% resistance, are likely to confer a combined effect of 96% overall resistance (Ehwaeti et al., 2000; Fuller et al., 2008). It is important to mention that the best transgenic event (17.1) in our study conferred 78.3% resistance to *M. incognita* in terms of reduced multiplication factor.

The adaptation of insects to the selective pressure of plant protease inhibitors is well known (Jongsma and Bolter, 1997; Zhu-Salzman and Zeng, 2015). Similar compensation for the inhibitory action of OC-IΔD86 could potentially occur in plant nematodes, considering that metallo and cysteine proteases are the most abundant proteolytic enzymes followed by serine, aspartic, and threonine proteases in *M. incognita* (Castagnone-Sereno et al., 2011). However, the majority of these proteinases are unlikely to play a role in proteolytic digestion in the intestine. One viable option that could counteract this potential problem, is a strategy that allows the construction of multi-cystatins or cystatin-containing hybrid protease inhibitors (van Wyk et al., 2016). A strategy in which two proteinase inhibitors were stacked together through translational fusion in *Arabidopsis* showed superior resistance to *H. schachtii* in comparison to either of the inhibitors alone (Urwin et al., 1998). On the contrary, transgenic banana expressing a dual construct of maize cystatin and a chemoreception disruptive synthetic peptide did not show additive resistance against *R. similis* (Roderick et al., 2012). Complete resistance to *G. pallida* (zero reproductive success) was achieved in a UK field trial when the natural partial resistance displayed by potato cultivars Sante and Maria Huanca was stacked with the transgenic resistance mediated by OC-IΔD86. In addition, transformed lines of cv. Sante with a cystatin transgene did not show susceptibility to virulent populations of *G. pallida* which otherwise usually break the natural resistance of Sante (Urwin et al., 2003). A similar strategy may be deployed for eggplant by combining the OC-IΔD86-mediated resistance and partial natural resistance conferred by *S. torvum* (Bagnaresi et al., 2013) to *M. incognita*.

## Conclusion

Our data demonstrate that cystatin overexpression in eggplant confers substantial resistance to *M. incognita* by inducing deleterious effects on nematode development and reproduction. The continuous delivery of bioactive molecules (proteinase inhibitors) to the feeding nematodes ensures the potential of this strategy to minimize RKN populations below the damage threshold level in eggplant. Although our data showed a satisfactory level of resistance in confinement, experimental trials are needed in RKN-infested fields to evaluate the effectiveness of the transgenic eggplant lines generated in our study. While considering the issues related to biosafety regulations of proteinase inhibitors, it is important to note that 10 mg daily consumption of recombinant OC-IΔD86 protein per kg body weight for consecutive 28 days did not cause any toxicity in mammals (Atkinson et al., 2004b). Further, transgenic potato expressing *OC-IΔD86* gene did not have any adverse effect on soil organisms in the potato rhizosphere (Cowgill and Atkinson, 2003; Cowgill et al., 2004) nor did it pose any substantial environmental threat to non-target above-ground organisms (Cowgill et al., 2002). In addition, transgenic expression of *OC-IΔD86* in potato roots did not perturb the free-living soil nematode communities in the field (Green et al., 2012). Therefore, improving nematode resistance in crop plants using cystatins and pyramiding with other proteinase inhibitors is a promising alternative to increase the global agricultural output.

## Author Contributions

PP carried out all the experiments, TD contributed in data analysis and writing the MS. NT helped in eggplant transformation and nematode bioassay experiments. CL made the cystatin construct and assisted with interpretation of the data, CL and PU critically revised the manuscript, UR conceived the experiments and wrote the manuscript. All authors read and approved the final manuscript.

## Conflict of Interest Statement

The authors declare that the research was conducted in the absence of any commercial or financial relationships that could be construed as a potential conflict of interest.

## Footnotes

1 http://faostat3.fao.org

2 http://www.isaaa.org/resources/publications/pocketk/48/default.asp
