# Supplementary material for: Expression of a Cystatin Transgene in Eggplant Provides Resistance to Root-knot Nematode, Meloidogyne incognita
Source: Front Plant Sci. 2016 Jul 28;7:1122. doi: 10.3389/fpls.2016.01122 (PMC4963396; doi:10.3389/fpls.2016.01122)

Supplementary Figure S1. Transformation of eggplant (cv. Pusa Purple Long) with OCI-ΔD86 construct and generation of transgenic lines. **A.** Seedling raised in MS medium, **B.** Pre-cultivation of leaf discs, **C.** Co-cultivation with *Agrobacterium*, **D.** Callus initiation of the explants, **E.** Callus differention, **F.** Shoot induction in the explant, **H.** Root induction in the explant, **I.** T_0 plants in pot, **J.** Flowering in T_0 plants, **K.** Fruiting after self-pollination. **L.** T_1 seeds

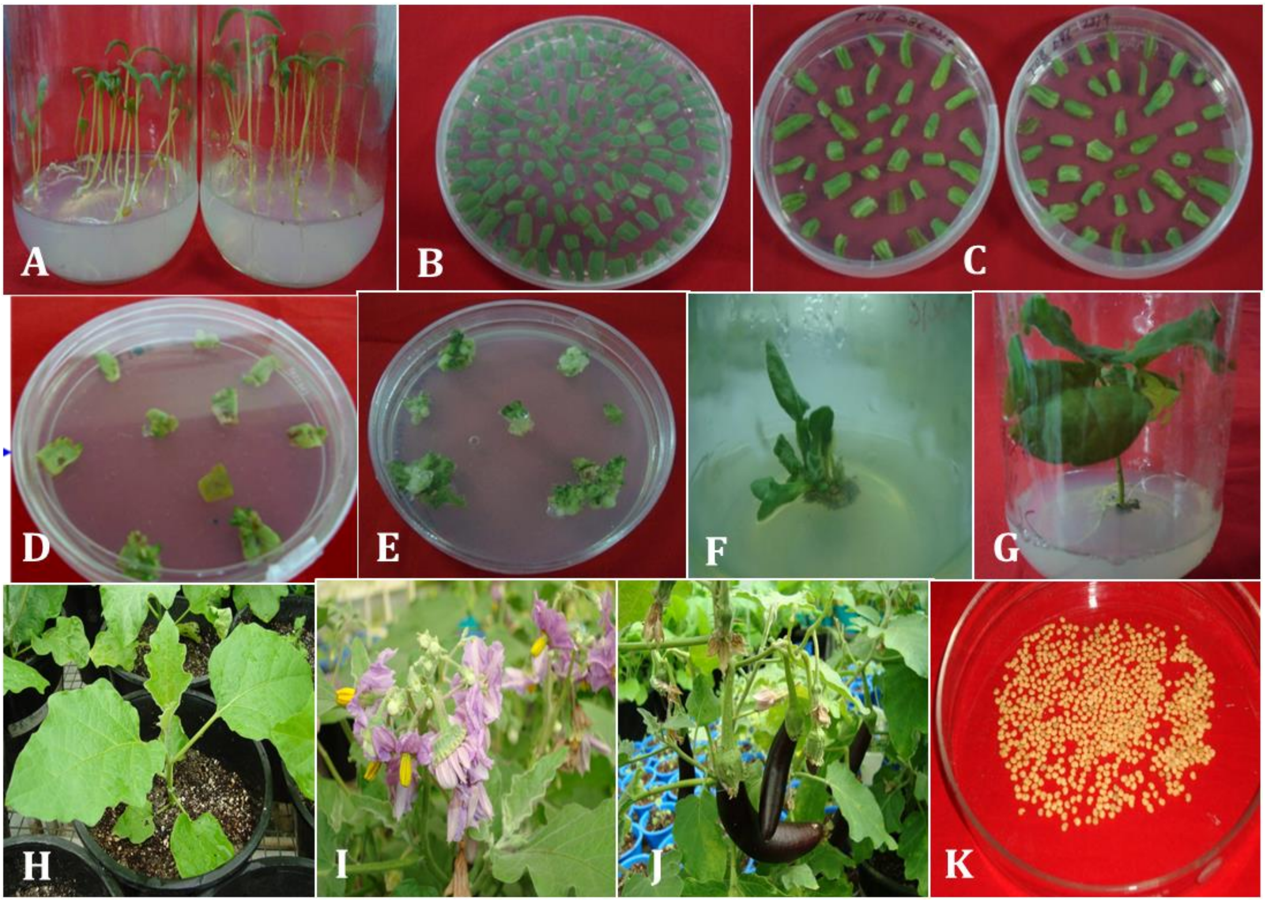

Supplementary Figure S2. Different PCR-positive T_0, T_1 and T_2 events of eggplant containing *OC-IΔD86* (256 bp) and *nptII* (750 bp) transgenes. M - 100 bp marker, UC – untransformed or wild type eggplant, +C – positive control.

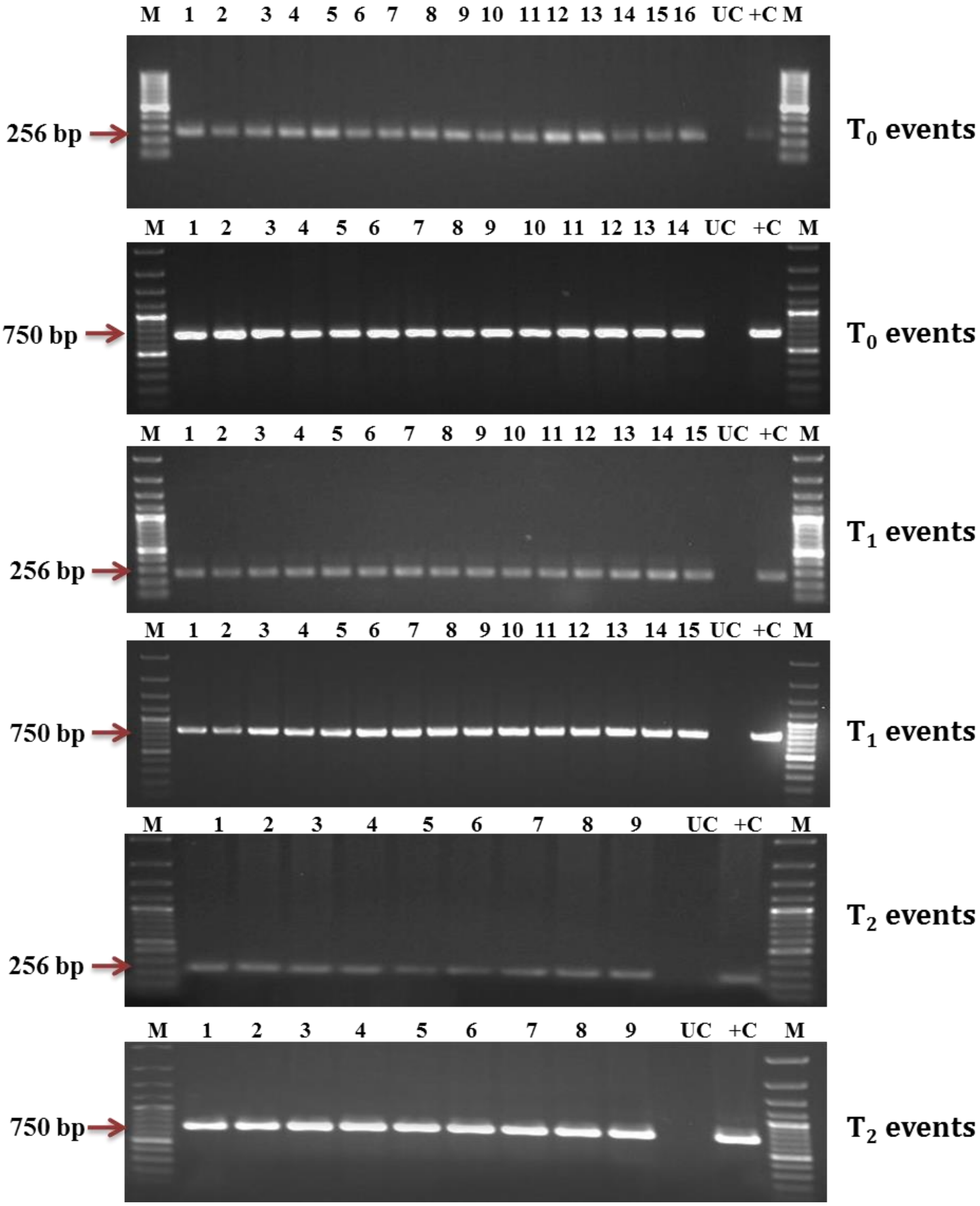

Supplementary Figure S3. Southern blot for T_2 eggplant transformants containing *OC-IΔD86* transgene. Progeny plants for event 9 (9.2 and 9.4) and 15 (15.2) exhibited double copy insertion of *OC-IΔD86* transgene. Progeny plants for event 1 (1.1 and 1.2) and all plants for event 13 and 17 showed single copy integration pattern. Untransformed control (UC) and wild type (WT) plants did not show any hybridization signal.

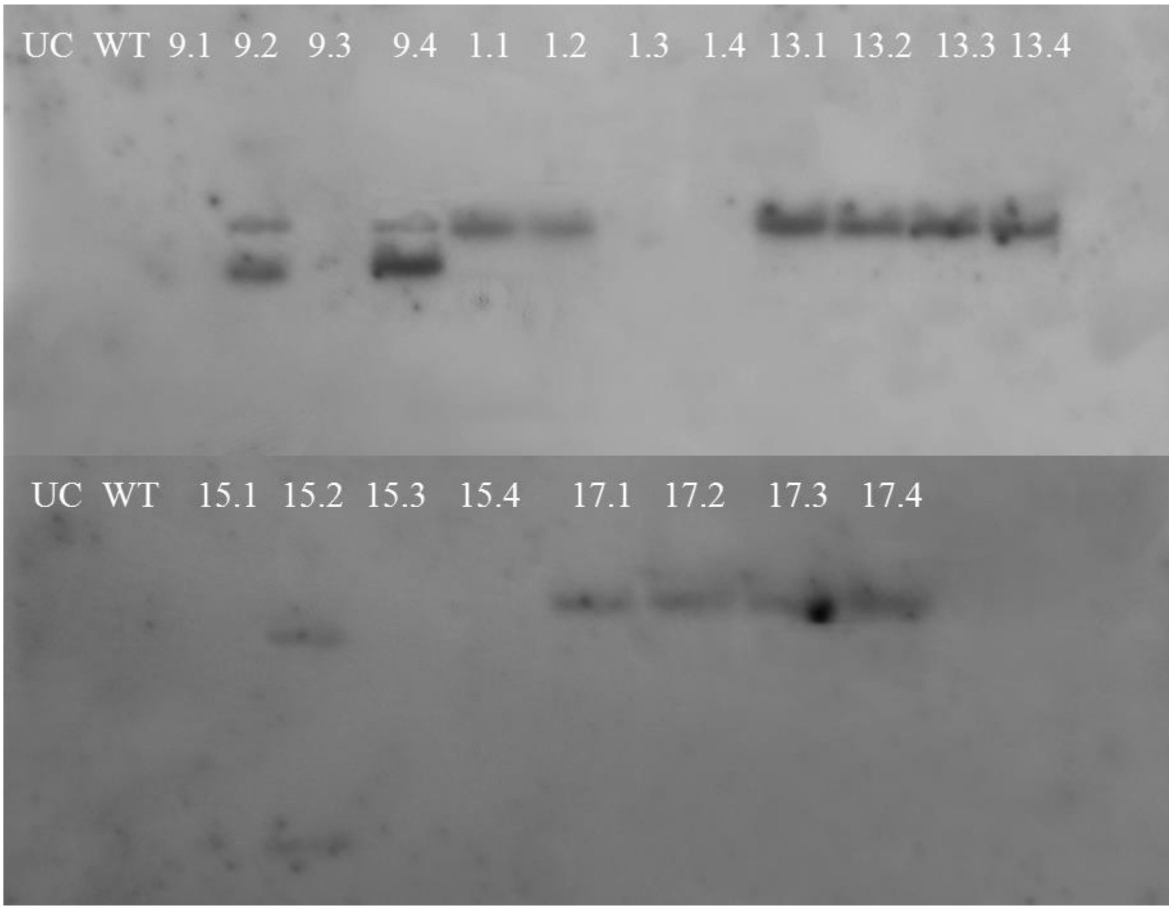

Supplementary Figure S4. Quantitative measurement of OC-IΔD86 in T_2 eggplant transformants through ELISA. **(A)** Standard curve displays absorbance calibration values for control samples spiked with synthesized OC-IΔD86. **(B)** Amount of OC-IΔD86 in events 1.1, 13.1, 17.1, 9.2 and 15.2. OC-IΔD86 could not be detected in wild type plants. % TSP values are presented as mean ± SD.

(A)

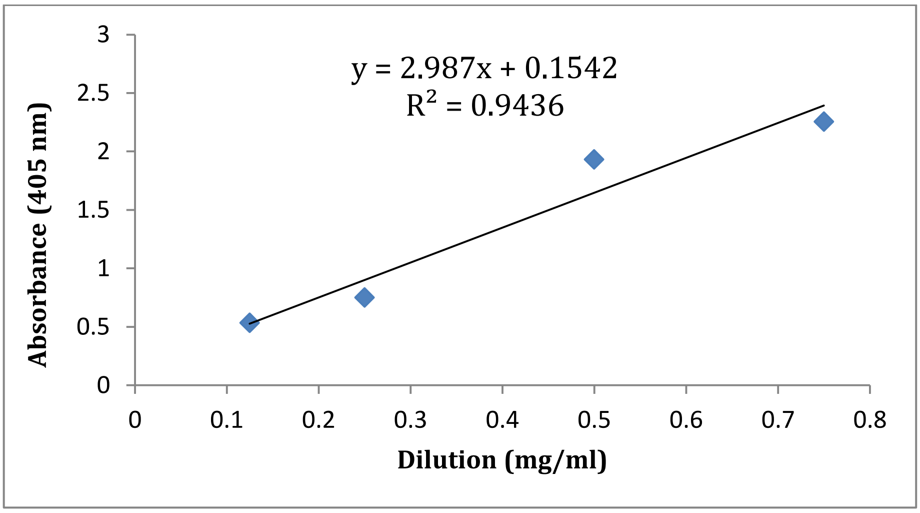

(B)

**Event number**	**Final concentration of OC-IΔD86 (mg/ml)** = $(Absorbance_{405nm}-0.154)/2.987$	**Amount of OC-IΔD86 (µg)** = initial sample volume (0.02 ml) × final concentration ×1000	**TSP (%)** = [amount of OC-IΔD86/ amount of total protein (5 µg)]×100
1.1	0.000375	0.0075	0.15 ± 0.04
13.1	0.0003	0.006	0.12 ± 0.03
17.1	0.00035	0.007	0.14 ± 0.02
9.2	0.000225	0.0045	0.09 ± 0.01
15.2	0.00025	0.005	0.10 ± 0.02

Supplementary Figure S5. (A) Root morphology and (**B)** Fresh weight of roots of wild type (WT) plants grown on non-selective medium and T_2 (1.1, 13.1, 17.1, 9.2 and 15.2) events of eggplant at 15 days post germination in selection medium. For each line six replicates were recorded.

A.

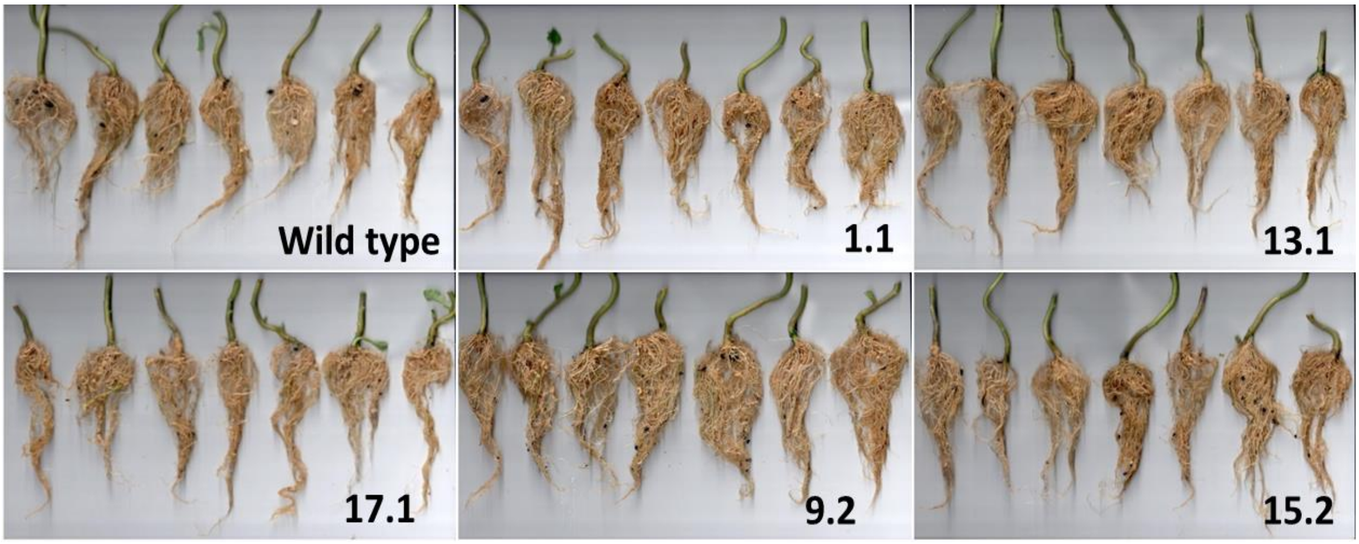

B.

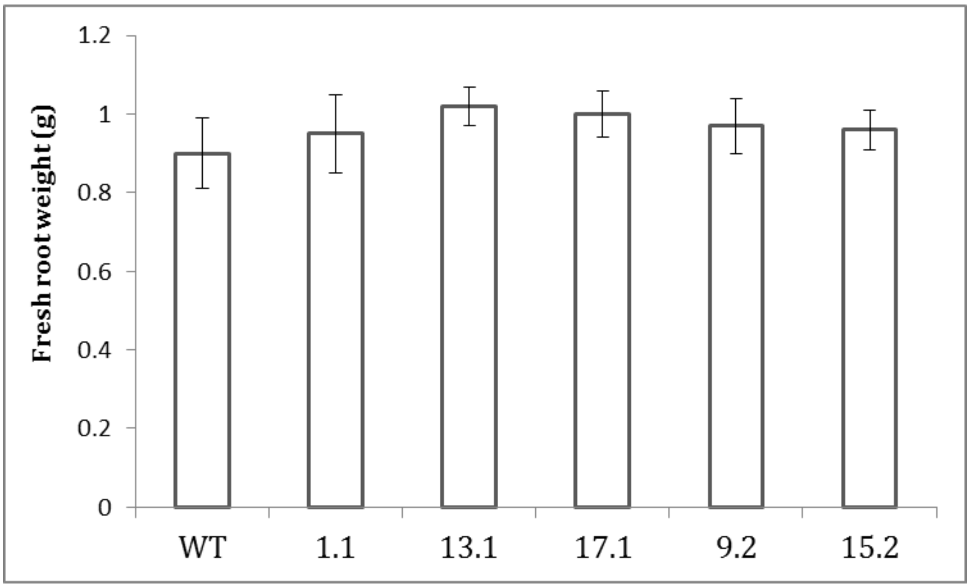

Supplementary Figure S6. Penetration success of *M. incognita* J2 in wild type (WT) and T_2 (1.1, 13.1, 17.1, 9.2 and 15.2) events of eggplant at 2 DPI.

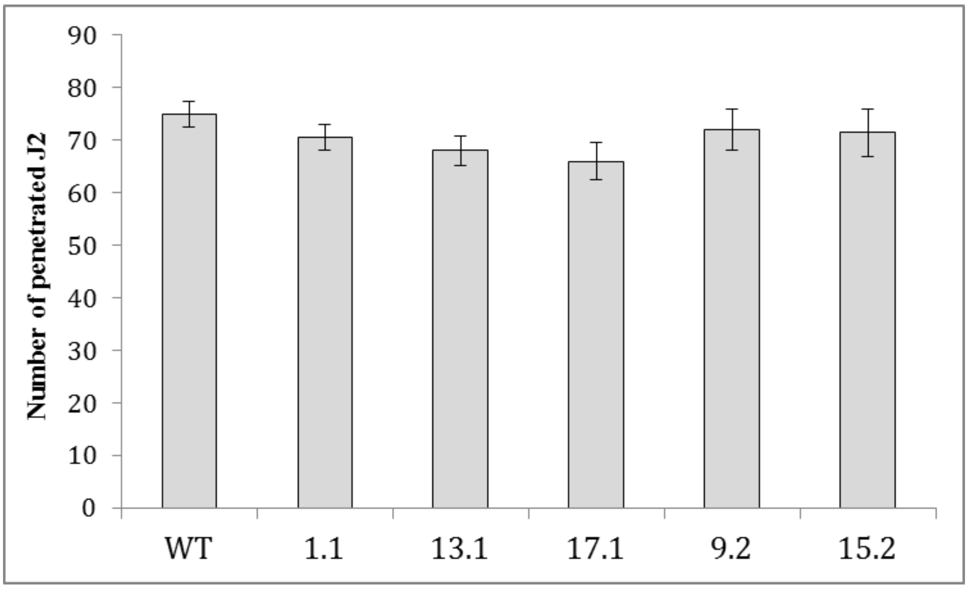

Supplement: Supplementary file 1 [file Presentation_1.PDF]
